# Association of the urinary sodium to urinary specific gravity ratio with metabolic syndrome in Korean children and adolescents: The Korea National Health and Nutrition Examination Survey 2010-2013

**DOI:** 10.1371/journal.pone.0189934

**Published:** 2017-12-18

**Authors:** Cheol Hwan So, Hwal Rim Jeong, Young Suk Shim

**Affiliations:** 1 Department of Pediatrics, Wonkwang University School of Medicine, Iksan, Republic of Korea; 2 Department of Pediatrics, Hallym University College of Medicine, Seoul, Republic of Korea; The University of Tokyo, JAPAN

## Abstract

**Objectives:**

This study aimed to evaluate the association between sodium intake and metabolic syndrome (MetS) in Korean boys.

**Methods:**

A total of 1,738 boys aged 10–18 years were included in this study from the Korea National Health and Nutrition Examination Survey (KNHANES) during the years 2010–2013. Sodium intake was assessed using the urinary sodium excretion to urinary specific gravity ratio (U-Na to U-SG ratio).

**Results:**

The median U-Na to U-SG ratio was 133.27 mmol/L (interquartile range: 95.66–178.50 mmol/L). Significant positive associations were found between the U-Na to U-SG ratio and the TG (*P* = 0.001 for trend) and TG concentrations, and these concentrations were significantly higher in boys with a U-Na to U-SG ratio in the highest quartile compared with those with a ratio in the lowest (*P* = 0.001) and second (*P* = 0.033) quartiles, as demonstrated through analysis of covariance (ANCOVA) after adjustment for possible confounders, including age, BMI standard deviation score, ferritin, vitamin D, house income, smoking, alcohol intake, physical activity, season, total intake, total energy intake, protein intake, fat intake, carbohydrate intake, and water intake. Significant inverse associations were found for the U-Na to U-SG ratio with the HDL-C (*P* = 0.033 for trend) and HDL-C levels, and these values were significantly lower in boys with a ratio in the highest quartile compared with those with a ratio in the second quartile (*P* = 0.020), as demonstrated through an ANCOVA. Although the trends did not reach statistical significance, a higher U-Na to U-SG ratio tended to be associated with higher SBP (*P* = 0.086 for trend), DBP (*P* = 0.063 for trend), and glucose levels (*P* = 0.099 for trend), as illustrated through ANCOVA. Boys with a ratio in the highest quartile exhibited a 1.73-fold increased risk for elevated TG (95% CI, 1.19–2.51) and a 2.66-fold increased risk for MetS (95% CI, 1.11–6.35) compared with those with a ratio in the lowest quartile, as demonstrated through multivariate logistic regression analyses after adjusting for confounders.

**Conclusions:**

Our results suggest that high sodium intake may be significantly independently associated with MetS in Korean boys aged 10–18 years.

## Introduction

According to the 2013 Korea School Health Examination, the prevalence of obesity in Korean children and adolescents increased from 13.2% in 2009 to 15.3% in 2013 [1]. The high prevalence of childhood and adolescent obesity has been associated with increased adult obesity [2] and obesity-related complications, such as insulin resistance [3], hypertension [4], dyslipidemia [5], metabolic syndrome (MetS) [6], and type 2 diabetes mellitus (T2DM) [7]. According to the modified NCEP-ATP III for children and adolescents, a recent Korean study revealed that the prevalence of MetS is 5.8% and 5.5% in boys and girls aged with 10–18 years, respectively [8]. MetS is related to cardiovascular disease, cerebrovascular disease, kidney disease, and type 2 diabetes mellitus (T2DM) [9]. This constellation of cardiometabolic risk factors is considered to be modifiable, and the identification of children and adolescents with MetS is necessary because age-specific interventions can help improve their condition.

High sodium intake has become one of the major problems in the healthcare field throughout the world. High sodium intake is associated with increased blood pressure in children [10], adolescents [10] and adults [11], and high sodium intake is thought to be related to MetS because elevated blood pressure is a component of MetS [12]. However, recent studies have indicated that high sodium intake is associated with other components of MetS [13–15]. There are reports that high sodium intake is related to obesity [13.14], and it has also been suggested that high sodium intake is associated with cardiometabolic risk factors, such as dyslipidemia, insulin resistance, and metabolic syndrome, in adults [15]. However, no previous study has demonstrated a link between high sodium and insulin resistance-related diseases in children and adolescents. In addition, high salt intake is modifiable as part of a medical intervention.

In the current study, we aimed to evaluate the association between sodium intake, which was assessed using the urinary sodium excretion to urinary specific gravity ratio (U-Na to U-SG ratio), and MetS in Korean boys aged 10–18 years. Through a nationwide survey, we also assessed whether this relationship was independent or whether the association was mediated by confounders.

## Materials and methods

### Subjects

**Fig 1:**
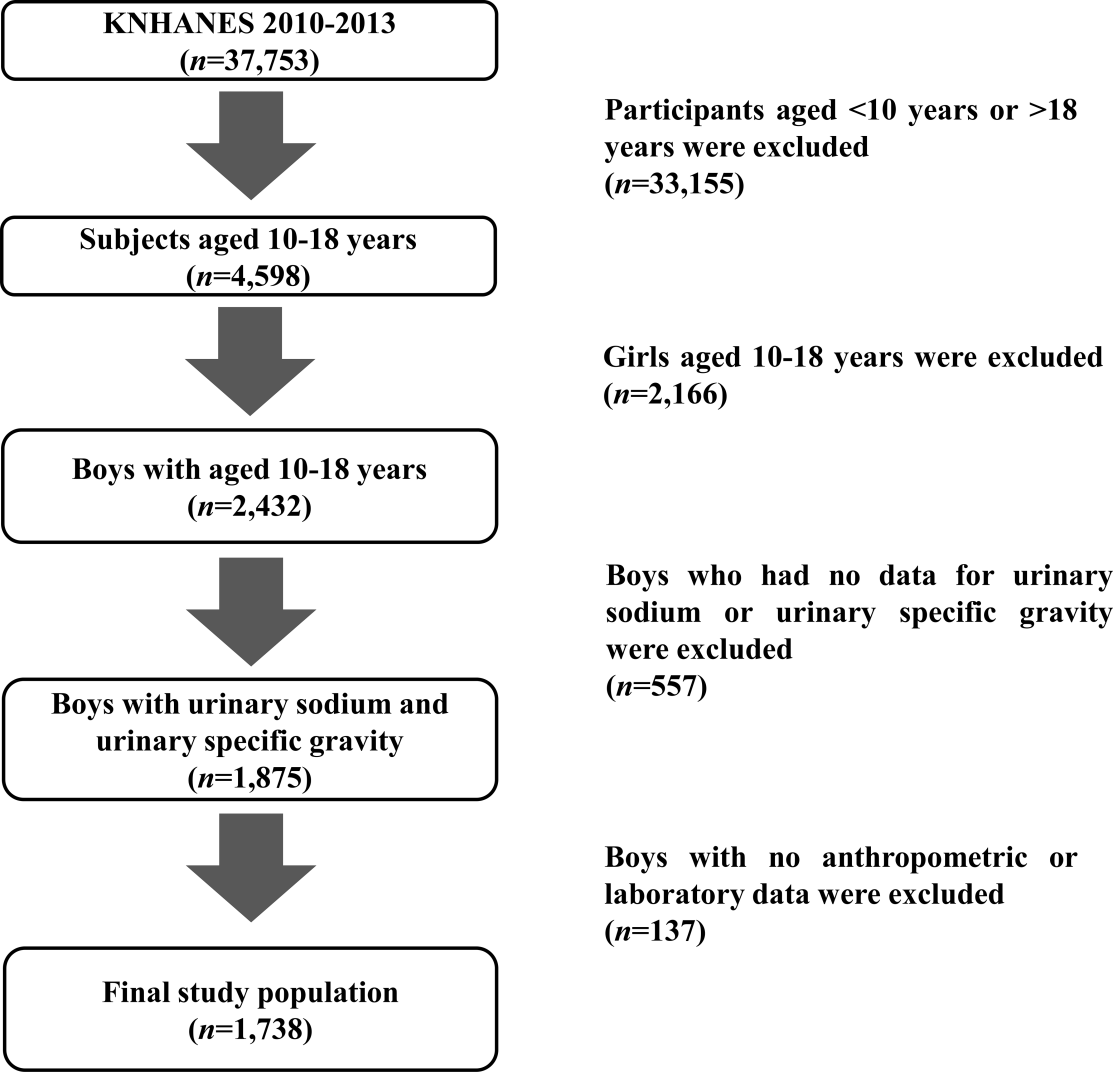
Flow chart of the study population. In total, 1,738 Korean boys were included in the present study.

This study was conducted using data from the Korea National Health and Nutrition Examination Survey (KNHANES) during the period 2010–2013. The KNHANES is a cross-sectional, nationwide and representative survey that is conducted regularly by the Division of Chronic Disease Surveillance, Korean Centers for Disease Control and Prevention [15]. The survey, which consists of a health questionnaire, health examination, and nutritional assessment, uses a stratified, multistage probability sampling design for the selection of household units. Details of the KNHANES have been described previously [16]. The 37,753 subjects in the KNHANES during the years 2010–2013 included 4,598 children and adolescents aged 10 to 18 years (Fig 1). Boys exhibited higher levels of sodium intake and excretion than girls in preliminary analyses. Additionally, the overall prevalence of MetS in girls was significantly lower than that in boys in our analyses. A total of 2,432 boys were included in our study, of which 1,875 underwent laboratory examination, including measurements of urinary sodium (U-Na) and urinary specific gravity (U-SG); 137 boys who did not complete the anthropometrical examination were excluded. As a result, 1,738 participants were included in this study. The database is available to the public at the KNHANES website (http://knhanes.cdc.go.kr) [17]. Because the dataset did not provide any personal information and informed consent was provided by all of the KNHANES participants, this study was exempted by the institutional review board from needing to obtain participant consent.

### Measurements

Anthropometric assessments using standard methods were conducted by a trained expert. In brief, the height and body weight were assessed to the nearest 0.1 cm using Seca 225 (Seca, Hamburg, Germany) and 0.1 kg using GL-6000-20 (G-tech, Seoul, Korea), respectively. The body mass index (BMI) was determined as the weight (kg)/square of height (m^2^). The waist circumference (WC) was measured at the midline between the lower rib margin and iliac crest to the nearest 0.1 cm. The standard deviation scores (SDSs) for the height, weight, BMI and WC were used because they were not evenly distributed between various age groups. The height SDS, weight SDS, BMI SDS and WC SDS were determined through LMS methods using 2007 Korean reference data. The systolic blood pressure (SBP, mmHg) and diastolic blood pressure (DBP, mmHg) were determined three times from the right upper arm using a calibrated sphygmomanometer (Baumanometer Desk model 0320, Baum, NY, USA) and an appropriately sized cuff. The measurements were taken at 2-minute intervals. The mean of the last two values was then used for the analysis.

Random samples from blood and urine were collected year-round after the participants had fasted for at least 8 hours, and these were immediately processed, refrigerated, and transported to a central laboratory (NeoDin Medical Institute, Seoul, Korea) for analysis within 24 hours. Routine biochemistry tests, including analyses of the levels of total cholesterol (T-C), high-density lipoprotein cholesterol (HDL-C), triglycerides (TG), glucose, U-Na and U-SG, were measured enzymatically using a Hitachi 7600 automatic analyzer (Hitachi, Tokyo, Japan). LDL cholesterol was determined with Friedewald’s equation [18]. Serum ferritin and vitamin D were determined through an immunoradiometric assay using a 1470 Wizard Gamma Counter (Perkin-Elmer, Turku, Finland).

### Collection of data for lifestyle-related and metabolic parameters

Smoking, alcohol intake, physical activity, and household income were included in this study as lifestyle-related parameters. Smokers were defined as individuals who smoked more than a total of five packs worth of cigarettes throughout their life and were divided into the following two groups: smoker and non-smoker. Alcohol intake was defined as drinking at least two alcoholic beverages/month during the previous year and was divided into the following two groups: drinker and non-drinker. Physical activity was defined as meeting at least one of the following three criteria: (i) intense physical activity for 20 minutes at least three days/week, (ii) moderate physical activity for 30 minutes at least five days/week, or (iii) walking for 30 minutes at least five days/week. Physical activity was also divided into the following two groups: exercise or no exercise. Household income was reported in quartiles and was categorized into the following two groups: lowest quartile and at least second quartile. The Republic of Korea is located in a temperate region with four seasons, which are categorized as spring (March to May), summer (June to August), fall (September to November), and winter (February and December).

The dietary intake of nutrients, including total intake, total energy intake, total protein, total fat, total carbohydrates, and water intake, was assessed by a trained nutritionist using 24-hour recall. The data regarding the general participant characteristics were obtained from the KNHANES.

### Definition

MetS and its components were defined according to modified criteria of the National Cholesterol Education Program Adult Treatment Panel III (NCEP ATP III) [19]. Elevated WC was defined as a WC greater than or equal to the 90th percentile for age and gender. Elevated BP was defined by an SBP or DBP greater than or equal to the 90th percentile for age, gender, and height according to 2007 Korean growth charts [20] or current administration of antihypertensive drugs. Elevated glucose was defined as fasting glucose concentrations greater than or equal to 110 mg/dL or a previous diagnosis of T2DM. T2DM was diagnosed in children and adolescents who met at least one of the following three categories: (i) subjects who self-reported their disease using a questionnaire comprising questions with yes or no answers, (ii) children and adolescents currently using medications or receiving insulin to manage T2DM, or (iii) participants with a fasting glucose level of at least 126 mg/dL during the national survey. Elevated TG was defined as serum TG concentrations greater than or equal to 110 mg/dL or current administration of drugs for dyslipidemia, whereas reduced HDL-C was defined as levels of serum HDL-C less than 40 mg/dL. MetS was defined as having at least three of the following five criteria: (i) elevated WC, (ii) elevated BP, (iii) elevated glucose, (iv) elevated TG, and (v) reduced HDL-C.

### Statistical analyses

All analyses were conducted using SPSS software for Windows (SPSS version 23.0, IBM SPSS Inc., Chicago, IL, USA). The participants were divided into four groups according to their U-Na to U-SG ratio: (i) lowest quartile group (Q1), (ii) second quartile group (Q2), (iii) third quartile group (Q3), and (iv) highest quartile group (Q4). Normally distributed variables are presented as the means ± standard errors (SEs), whereas categorical variables are presented as percentages (%). Differences in categorical variables and normally distributed variables were analyzed using chi-square tests and analysis of variance (ANOVA) according to the U-Na to U-SG ratio. Adjusted means for MetS components, including WC SDS, SBP, DBP, glucose, HDL-C, and TG, were calculated through an analysis of covariance (ANCOVA) with Bonferroni’s *post hoc* test after adjustment for possible confounding factors, such as age, BMI SDS, ferritin, vitamin D, house income, smoking, alcohol intake, physical activity, season, total intake, total energy intake, protein intake, fat intake, carbohydrate intake, and water intake according to the U-Na to U-SG ratio, which was assessed as a surrogate for sodium intake and divided into quartiles. To investigate the associations of the U-Na to U-SG ratio with MetS and its components, a multivariate logistic regression analysis was conducted after adjusting for the previously described confounders, and the corresponding odds ratios (ORs) and 95% confidence intervals (95% CIs) were determined. The ORs for MetS components and MetS were determined according to the U-Na to U-SG ratio (divided into quartiles), with the lowest quartile serving as a reference. Trends across quartiles were assessed for each serum ferritin quartile as continuous variables in the multivariate logistic regression models. All significances were analyzed using a two-tailed method, and a *P* value <0.05 was considered to indicate statistical significance.

## Results

### Clinical characteristic of study participants according to the U-Na to U-SG ratio

The mean age of the study participants was 13.79 ± 0.06 years, and the median U-Na to U-SG ratio was 133.27 mmol/L, with an interquartile range (IQR) of 95.66–178.50 mmol/L. The median U-Na to U-SG ratios in the lowest, second, third and highest quartiles were 71.99 mmol/L (13.92–95.66 mmol/L), 115.76 ng/mL (95.66–133.27 mmol/L), 155.64 mmol/L (133.27–178.50 mmol/L), and 212.33 mmol/L (178.50–463.80 mmol/L), respectively. The clinical characteristics of the study population are shown in **Table 1**. The subjects with a U-Na to U-SG ratio in the highest quartile tended to have increased mean values for the weight SDS (*P*<0.001), BMI SDS (*P*<0.001), WC SDS(*P*<0.001), SBP (*P* = 0.011), DBP(*P* = 0.013), glucose levels (*P* = 0.003), T-C levels (*P* = 0.034), TG levels (*P*<0.001), ferritin levels (*P* = 0.010), U-SG (*P*<0.001), and U-Na (*P*<0.001), respectively. Boys with a U-Na to U-SG ratio in the higher quartiles were more likely to have a lower house income (*P* = 0.004) and a lower mean HDL-C level (*P* = 0.003).

**Table 1:** Clinical characteristic of the study participants according to the urinary sodium to urinary specific gravity ratio in Korean boys aged 10–18 years (*n* = 1,738).

	Urinary sodium to urinary specific gravity ratio (mmol/L)				
	Q1	Q2	Q3	Q4	
	n = 434	n = 435	n = 435	n = 434	
	[<95.66]	[95.66–133.27]	[133.27–178.50]	[≥178.50]	*P*
Age (years)	13.90 ± 0.12	13.73 ± 0.12	13.72 ± 0.12	13.82 ± 0.12	0.697
Height SDS	0.38 ± 0.05	0.52 ± 0.05	0.53 ± 0.05	0.47 ± 0.05	0.147
Weight SDS	0.02 ± 0.05	0.16 ± 0.05	0.28 ± 0.05	0.38 ± 0.06	<0.001
BMI SDS	-0.19 ± 0.05	-0.08 ± 0.05	0.04 ± 0.05	0.21 ± 0.05	<0.001
WC SDS	-0.44 ± 0.05	-0.31 ± 0.05	-0.19 ± 0.05	-0.01 ± 0.06	<0.001
SBP (mmHg)	106.93 ± 0.51	107.57 ± 0.59	107.49 ± 0.52	109.33 ± 0.54	0.011
DBP (mmHg)	66.77 ± 0.47	66.83 ± 0.47	66.32 ± 0.46	67.87 ± 0.46	0.013
Glucose (mg/dL)	89.16 ± 0.30	89.45 ± 0.31	90.04 ± 0.27	90.64 ± 0.31	0.003
T-C (mg/dL)	151.83 ± 1.23	157.11 ± 1.36	154.24 ± 1.24	155.93 ± 1.53	0.034
HDL-C (mg/dL)	53.26 ± 0.50	54.24 ± 0.52	52.98 ± 0.51	51.58 ± 0.49	0.003
TG (mg/dL)	75.87 ± 1.96	80.60 ± 2.21	85.31 ± 2.54	93.46 ± 3.20	<0.001
LDL-C (mg/dL)	83.40 ± 1.03	86.75 ± 1.16	84.39 ± 1.07	86.23 ± 1.37	0.145
Ferritin (ng/mL)	53.44 ± 1.91	51.00 ± 1.64	48.84 ± 1.40	45.94 ± 1.56	0.010
Vitamin D (ng/mL)	17.88 ± 0.29	17.93 ± 0.27	18.30 ± 0.28	17.92 ± 0.26	0.672
Urinary specific gravity (mmol/L)	1.02 ± 0.00	1.02 ± 0.00	1.02 ± 0.00	1.02 ± 0.00	<0.001
Urinary sodium (mmol/L)	70.94 ± 0.93	117.92 ± 0.55	159.18 ± 0.64	224.43 ± 1.87	<0.001
Urinary sodium to urinary specific gravity ratio	69.50 ± 0.90	115.27 ± 0.54	155.44 ± 0.62	219.26 ± 1.83	<0.001
Season					0.576
Spring	84 (19.4%)	96 (22.1%)	78 (17.9%)	72 (16.6%)	
Summer	118 (27.2%)	115 (26.4%)	109 (25.1%)	121 (27.9%)	
Autumn	110 (25.3%)	105 (24.1%)	120 (27.6%)	126 (29.0%)	
Winter	122 (28.1%)	119 (27.4%)	128 (29.4%)	115 (26.5%)	
House income ≤lowest quartile (%)	39 (9.0%)	54 (12.4%)	45 (10.3%)	72 (16.6%)	0.004
Smoking (%)	41 (9.4%)	34 (7.8%)	38 (8.7%)	43 (9.9%)	0.723
Alcohol intake (%)	44 (10.1%)	42 (9.7%)	41 (9.4%)	42 (9.7%)	0.988
Physical activity (%)	272 (62.7%)	277 (63.7%)	276 (63.4%)	296 (68.2%)	0.312
Total intake (g/day)	1455.72 ± 33.66	1453.13 ± 30.85	1412.77 ± 31.97	1400.72 ± 34.32	0.533
Total energy intake (kcal/day)	2275.36 ± 43.76	2283.16 ± 42.33	2288.13 ± 43.19	2282.06 ± 47.83	0.998
Protein intake (g/day)	81.27 ± 2.24	81.30 ± 1.92	83.13 ± 1.94	81.67 ± 2.01	0.907
Fat intake (g/day)	57.90 ± 1.96	57.22 ± 1.65	60.68 ± 2.07	56.70 ± 1.75	0.445
Carbohydrate intake (g/day)	355.57 ± 6.26	359.72 ± 6.65	352.24 ± 6.15	357.28 ± 7.34	0.879
Sodium intake (g/day)	4.82 ± 0.12	4.36 ± 0.11	4.74 ± 0.13	4.70 ± 0.15	0.016
Water intake (g/day)	933.21 ± 26.48	932.37 ± 24.29	890.92 ± 25.09	878.89 ± 26.68	0.309

The data are shown as the means ± SEs (standard errors).

BMI; body mass index, SDS; standard deviation score, WC; waist circumference, SBP; systolic blood pressure, DBP; diastolic blood pressure, HOMA-IR; homeostatic model assessment of insulin resistance, QUICKI; quantitative insulin sensitivity check index, T-C; total cholesterol, HDL-C; high-density lipoprotein cholesterol, TG; triglyceride, LDL-C; low-density lipoprotein cholesterol.

### Adjusted means for MetS components according to the U-Na to U-SG ratio

The adjusted means for MetS components were calculated through an ANCOVA after adjustment for possible confounding factors, including age, BMI SDS, ferritin, vitamin D, house income, smoking, alcohol intake, physical activity, season, total intake, total energy intake, protein intake, fat intake, carbohydrate intake, and water intake according to the U-Na to U-SG ratio (divided into quartiles). The adjusted means for MetS components according to the U-Na to U-SG ratio are presented in **Table 2**. Significant inverse associations were found for observed between the U-Na to U-SG ratio and HDL-C (*P* = 0.033 for trend). Significantly lower HDL-C levels were detected in the boys in Q4 than in the boys in Q2 (*P* = 0.020). A significant positive association was observed between the U-Na to U-SG ratio and TG (*P* = 0.001 for trend). The TG concentrations were significantly higher in boys in Q4 compared with the boys in Q1 (*P* = 0.001) and Q2 (*P* = 0.033). Although the trends did not reach statistical significance, a higher U-Na to U-SG ratio tended to be associated with higher SBP (*P* = 0.086 for trend), DBP (*P* = 0.063 for trend), and glucose (*P* = 0.099 for trend) values.

**Table 2:** Adjusted means for metabolic syndrome (MetS) components according to the urinary sodium to urinary specific gravity ratio in Korean boys aged 10–18 years (*n* = 1,738).

	Urinary sodium to urinary specific gravity ratio				
	Q1	Q2	Q3	Q4	*P* for trend
WC SDS	-0.30 ± 0.02	-0.25 ± 0.02	-0.25 ± 0.02	-0.22 ± 0.02	0.173
SBP (mmHg)	106.95 ± 0.50	107.72 ± 0.50	107.47 ± 0.51	108.76 ± 0.51	0.086
DBP (mmHg)	66.15 ± 0.45	66.93 ± 0.44	66.26 ± 0.45	67.69 ± 0.46	0.063
Glucose (mg/dL)	89.56 ± 0.31	89.67 ± 0.30	90.12 ± 0.31	90.55 ± 0.32	0.099
HDL-C (mg/dL)	53.11 ± 0.52	54.29 ± 0.51	52.98 ± 0.53	52.14 ± 0.53^b^	0.033
TG (mg/dL)	77.89 ± 2.66	82.23 ± 2.63	85.29 ± 2.71	92.79 ± 2.72^a^^,^^b^	0.001

The data are shown as the means ± SEs (standard errors).

WC; waist circumference, SDS; standard deviation score, SBP; systolic blood pressure, DBP; diastolic blood pressure, HDL-C; high-density lipoprotein cholesterol, TG; triglyceride.

Adjusted means for WC SDS, SBP, DBP, glucose, HDL-C, and TG were determined after adjustment for age, body mass index (BMI) SDS, ferritin, vitamin D, house income, smoking, alcohol intake, physical activity, season, total intake, total energy intake, protein intake, fat intake, carbohydrate intake, sodium intake, and water intake through an analysis of covariance (ANCOVA) according to the urinary sodium to urinary specific gravity ratio.

^a^: P<0.05 vs. the first quartile

^b^: P<0.05 vs. the second quartile

^c^: P<0.05 vs. the third quartile.

### Adjusted ORs of MetS and its components according to the U-Na to U-SG ratio

The overall prevalence of MetS was 4.2% in this study. The prevalences of MetS according to a U-Na to U-SG ratio in the lowest, second, third and highest quartiles were 2.8%, 3.7%, 3.7%, and 6.7%, respectively (*P* = 0.024). The risks of MetS components and MetS according to the U-Na to U-SG ratio (divided into quartiles) were assessed through a multivariate logistic regression analysis after controlling for the abovementioned possible confounders. **Table 3** shows the adjusted ORs for MetS and its components according to the U-Na to U-SG ratio. Boys with a U-Na to U-SG ratio in the highest quartile had a 1.73-fold increased risk for elevated TG (95% CI, 1.19–2.51) and a 2.66-fold increased risk for MetS (95% CI, 1.11–6.35) compared with those with a ratio in the lowest quartile. Additionally, significant positive linear associations were observed for the quartiles of the U-Na to U-SG ratio with elevated TG (*P* = 0.004 for trend) and MetS (*P* = 0.039 for trend).

**Table 3:** Adjusted odds ratio (95% CI) of metabolic syndrome (MetS) and its components according to the urinary sodium to urinary specific gravity ratio in Korean boys aged 10–18 years (*n* = 1,738).

	Urinary sodium to urinary specific gravity ratio				
	Q1	Q2	Q3	Q4	*P* for trend
Elevated WC	Reference	0.97 (0.38–2.45)	0.35 (0.13–0.94)	0.72 (0.29–1.78)	0.249
Elevated BP	Reference	1.10 (0.79–1.52)	0.87 (0.62–1.22)	1.27 (0.91–1.76)	0.352
Elevated glucose	Reference	1.01 (0.13–7.58)	0.79 (0.10–5.99)	1.38 (0.23–8.43)	0.741
Reduced HDL-C	Reference	0.97 (0.57–1.65)	1.18 (0.70–1.99)	1.32 (0.79–2.21)	0.209
Elevated TG	Reference	1.34 (0.92–1.95)	1.44 (0.99–2.10)	1.73 (1.19–2.51)	0.004
MetS	Reference	1.74 (0.68–4.42)	1.28 (0.49–3.34)	2.66 (1.11–6.35)	0.039

WC, waist circumference; BP, blood pressure; HDL-C, high-density lipoprotein cholesterol; TG, triglyceride, MetS; metabolic syndrome.

The adjusted odds ratios (ORs) of MetS and its components were calculated through multivariate logistic regression analyses after adjustment for age, body mass index (BMI) standard deviation scores (SDS), ferritin, vitamin D, house income, smoking, alcohol intake, physical activity, season, total intake, total energy intake, protein intake, fat intake, carbohydrate intake, sodium intake, and water intake.

Elevated WC was defined as values greater than or equal to the 90th percentile for age and gender. Elevated BP was defined as SBP or DBP greater than or equal to the 90th percentile for age, gender, and height or current administration of antihypertensive drug. Elevated glucose was defined as glucose greater than or equal to 110 mg/dL or medication for diabetes. Elevated TG was defined as TG greater than or equal to 110 mg/dL or current administration of drugs for dyslipidemia. Reduced HDL-C was defined as HDL-C less than 40 mg/dL or current administration of drugs for dyslipidemia.

## Discussion

In the current nationally representative study, the boys with a U-Na to U-SG ratio (which was assessed as a surrogate for sodium intake) in the highest quartile exhibited increased mean weight SDS, BMI SDS, and WC SDS values. Covariance analyses revealed a significant positive association between the U-Na to U-SG ratio and TG and a significant inverse association between the U-Na to U-SG ratio and HDL-C in Korean boys aged 10–18 years after adjustment for possible confounders. An ANCOVA showed that a higher U-Na to U-SG ratio tended to be associated with higher SBP, DBP, and glucose values after controlling for possible confounders, but the trends did not reach statistical significance. After adjusting for confounders, multivariate logistic regression analyses revealed that boys with a U-Na to U-SG ratio in the highest quartile exhibited significantly increased risks for elevated TG and MetS compared with those with a ratio in the lowest quartile.

A previous study suggested that high sodium intake is related to MetS, which is characterized by a set of cardiometabolic risk factors, including central obesity, high blood pressure, impaired fasting glucose, and dyslipidemia (elevated triglyceride concentration and low HDL-C concentration) [21]. This relationship may be predominantly explained by previous findings that high sodium intake is closely linked to increased blood pressure [22]. A Brazilian study demonstrated that high sodium intake is associated with BP but not with other MetS components [12]. A study conducted by Hoffmann et al. in Venezuela found that higher urinary sodium is associated with obesity and higher BP but not dyslipidemia or fasting glucose [23]. However, there is evidence indicating that sodium intake is independently associated with insulin resistance-related diseases, such as dyslipidemia, metabolic syndrome and T2DM, in adults. The OPERA (Oulu Project Evaluating the Risk of Atherosclerosis) study found that high sodium intake is associated with MetS components, such as WC and high fasting glucose [24], and Baudrand et al. reported that a high sodium diet is associated with dyslipidemia in Chilean adults [15]. A recent Korean study demonstrated that the estimated 24-hour urine excretion of sodium, which was used as a surrogate of sodium intake, is associated with MetS and its components [25]. Some studies conducted in pediatric fields have indicated that high sodium intake is related to obesity and hypertension [15,26,27], but few studies have evaluated the association of sodium intake with MetS and its components with the exception of elevated BP. In this study, after adjusting for confounders, we performed multivariate logistic regression analyses and demonstrated that boys with a U-Na to U-SG ratio in the highest quartile exhibited significantly higher risks for elevated TG and MetS compared with those with a U-Na to U-SG ratio in the lowest quartile. Our results are in line with previous reports demonstrating that a high sodium intake is related to MetS.

The pathophysiology of the association between sodium intake and insulin resistance is not fully understood. A possible explanation is that high sodium intake is associated with obesity [28], which is predominantly related to insulin resistance, a key mechanism of MetS. It has been demonstrated that increased salt intake is related to stimulation of thirst and appetite and that there is a positive association between salt intake and sugar-sweetened soft drink consumption [29]. Accordingly, a high salt intake is thought to lead to high energy intake and obesity. An Australian study reported that dietary salt intake predicts total fluid consumption and sugar-sweetened beverage consumption, which are associated with obesity risk [30]. However, in the present study, the U-Na to U-SG ratio, a surrogate for sodium intake, was found to be significantly independently associated with MetS after controlling for possible confounding factors.

Other explanations should be considered. Animal studies have yielded evidence indicating that a high salt diet may be independently associated with fat metabolism and insulin resistance, which is a key mechanism of MetS. A high salt diet induces higher adiposity and adipocyte hypertrophy, although no significant changes in the rat’s body weight were detected [31]. In a rat model of MetS, salt restriction induced improvement in insulin resistance without reducing obesity [32]. An animal study suggested that insulin resistance induced by a high sodium diet is related to impaired insulin-stimulated microvascular recruitment [33]. In addition, human studies have indicated that high sodium intake may be independently associated with MetS and its components. A US study suggested that high sodium intake is independently positively associated with adiposity and inflammation in adolescents after adjusting for total energy intake and sugar-sweetened soft drink consumption [34]. It has been reported that high sodium intake is independently related to MetS components, including insulin resistance, dyslipidemia, and hypertension, even after adjusting for confounding variables including age, gender, and BMI [25,35]. Alternatively, a low dietary sodium intake reduces insulin secretion in humans independently of insulin resistance [36]. This study demonstrated that high sodium intake was significantly independently associated with TG and MetS, which is in line with the results from previous studies.

The gold standard for the assessment of sodium intake is urinary sodium excretion from 24-hour urine collection [37], which is more inconvenient than spot urine collection in clinical settings, and approximately 30% of individuals submit under-collections and underestimations of their actual sodium intake [38]. Spot urine samples can be used in large population studies. There are clinically applicable 24-hour sodium excretion equations using spot urinary sodium and urinary creatinine for adults [37,38]. In addition, population-based studies have validated the estimation of 24-hour urinary sodium secretion in adults [22,39]. However, no studies have assessed the medical formula using spot urinary sodium to estimate 24-hour urinary sodium for children and adolescents. A Korean study in adolescents showed that the U-Na to U-SG ratio may be used as a surrogate for sodium intake [35]. In the current study, we utilized the U-Na to U-SG ratio as a surrogate for sodium intake and showed a significant positive association between the U-Na to U-SG ratio and MetS. Nevertheless, a valid and reliable formula for estimating 24-hour urinary sodium using spot urine samples from children and adolescents should be investigated.

A potential limitation of the present study is its cross-sectional nature because causality cannot be proven. Second, we could not adjust the data for pubertal status, familial history of T2DM, or levels of several adipokines. Third, we analyzed the data for dietary intake of nutrients based on a nutritional survey using 24-hour dietary recall, but with dietary recall, many subjects are truly unaware of the amount and type of nutrients they have consumed [40], which may have affected the accuracy of the data, potentially leading to recall bias. However, this study was performed using data from a nationwide examination survey, and the dietary intake of nutrients was adjusted for as a confounder in the ANCOVA and multivariate logistic regression analyses. Finally, we could not adjust for various confounding factors that can influence sodium homeostasis, including past medical history and medications, such as diuretics and inhibitors of the renin–angiotensin system.

The present nationwide, population-based, cross-sectional study showed that the U-Na to U-SG ratio, as a surrogate for sodium intake, was significantly positively associated with TG and significantly inversely associated with HDL-C in Korean boys aged 10–18 years, as demonstrated through an ANCOVA after adjustment for possible confounders. Although the trends did not have statistical significance, a higher U-Na to U-SG ratio tended to be associated with higher SBP, DBP, and glucose values, as demonstrated through an ANCOVA after controlling for confounders. The highest quartile of the U-Na to U-SG ratio was significantly related to increased risks for elevated TG and MetS compared with the lowest quartile among Korean boys aged 10–18 years, as demonstrated through multivariate logistic regression analyses after adjusting for confounders. Our results suggest that high sodium intake, which is determined as the U-Na to U-SG ratio, may be significantly independently associated with MetS in Korean boys aged 10–18 years.
